# Longitudinal Trends in Blood Pressure Associated with The Changes in Living Environment Caused by the Great East Japan Earthquake: The Fukushima Health Management Survey

**DOI:** 10.3390/ijerph20010857

**Published:** 2023-01-03

**Authors:** Satomi Ikeda, Ai Ikeda, Tetsuya Ohira, Akira Sakai, Michio Shimabukuro, Masaharu Maeda, Hirooki Yabe, Masanori Nagao, Seiji Yasumura, Hitoshi Ohto, Kenji Kamiya, Takeshi Tanigawa

**Affiliations:** 1 Department of Public Health, Juntendo University Graduate School of Medicine, Tokyo 113-8421, Japan; 2 Faculty of International Liberal Arts, Juntendo University, Tokyo 113-8421, Japan; 3 Radiation Medical Science Center for the Fukushima Health Management Survey, Fukushima Medical University, Fukushima 960-1295, Japan; 4 Department of Epidemiology, Fukushima Medical University, Fukushima 960-1295, Japan; 5 Department of Radiation Life Sciences, Fukushima Medical University, Fukushima 960-1295, Japan; 6 Department of Diabetes, Endocrinology, and Metabolism, Fukushima Medical University, Fukushima 960-1295, Japan; 7 Department of Disaster Psychiatry, Fukushima Medical University, Fukushima 960-1295, Japan; 8 Department of Neuropsychiatry, Fukushima Medical University, Fukushima 960-1295, Japan; 9 Department of Public Health, Fukushima Medical University, Fukushima 960-1295, Japan; 10 Research Institute for Radiation Biology and Medicine, Hiroshima University, Hiroshima 739-8511, Japan

**Keywords:** changes in the living environment, stress, cardiovascular-related biomarker, blood pressure, longitudinal study, the Great East Japan Earthquake

## Abstract

The Great East Japan Earthquake occurred on 11 March 2011, forcing Fukushima Prefecture residents to change their living environment. Such sudden changes possibly have long-term effects on cardiovascular-related diseases. We therefore sought to identify temporal relationships between living environment changes and blood pressure levels over three years following the earthquake. Participants included 14,941 men and 21,533 women aged 16 years or older who answered self-administered questionnaires, including questions on living environment changes at baseline (2012). Blood pressure levels were measured each year from 2012 to 2015. Linear mixed-effects models were used to analyze associations between living environment changes and blood pressure levels. Men with changes in living environment (i.e., those living in shelters or in temporary housing, rental apartments, relatives’ houses, or others) showed significantly higher diastolic blood pressure levels than those who lived in their home at baseline (77.3 mmHg vs. 77.8 mmHg; *p* < 0.001). The time-dependent effect of diastolic blood pressure levels associated with living environment was not statistically significant, indicating a sustained difference in diastolic blood pressure associated with living environment changes at baseline after three years. The effect of living environment changes on diastolic blood pressure increment was also evident in men without antihypertensive medication use during the study period and in men who were current drinkers at baseline. There were no associations between living environment changes and diastolic blood pressure levels among women. Sudden changes in living environment due to the disaster had an impact on the long-term effects of higher diastolic blood pressure among middle-aged men.

## 1. Introduction

The Great East Japan Earthquake that occurred on 11 March 2011 in Japan was catastrophic. The earthquake and subsequent tsunami damaged the Fukushima Daiichi Nuclear Power Plant (NPP), releasing hazardous radiation into Fukushima Prefecture, particularly the coastal regions, and forcing more than 160,000 residents to evacuate the area. This natural disaster and the subsequent evacuation led to changes in living environments; loss of family, friends, and relatives; physical injuries; loss of property and jobs; disruption of social networks; and reduced dietary conditions, thereby inducing psychological stress in the evacuees [1,2,3]. Immediately after the Great East Japan Earthquake, most evacuees lived in evacuation shelters for several months. Approximately six months later, some evacuees were transferred to temporary housing, where only basic necessities were supplied by the local government [4]. The rebuilding of houses as part of the recovery plans took several years, forcing evacuees to live in temporary housing for extended periods [1]. Such sudden changes in the living environment cause high stress and anxiety, possibly promoting arteriosclerosis by activating the sympathetic nervous system [5] and abnormal glucose metabolism [6], which is deeply involved in the onset of lifestyle diseases and associated mortality [7,8,9,10,11,12,13,14]. In Fukushima Prefecture in particular, the effects of evacuation from the Fukushima Daiichi Nuclear Power Plant have been prolonged. A study with an average follow-up of 1.6 years from 2011 onwards reported an increase in mean blood pressure levels and hypertension prevalence immediately after the earthquake, especially among male evacuees [15]. Despite several studies comparing evacuees with non-evacuees [15,16], no previous longitudinal study has examined changes in blood pressure levels with changes in the living environment due to the earthquake. These living environment changes remain a factor inducing psychological distress following the earthquake.

Therefore, our present study investigated the association between sudden changes in the living environment due to the earthquake and changes in blood pressure levels after the earthquake in Fukushima Prefecture residents. We examined our *a priori* hypothesis that living environment changes might increase blood pressure levels. Moreover, we aimed to identify the temporal relationships between sudden changes in the living environment and systolic and diastolic blood pressure levels over the three years (2012–2015) following the earthquake.

## 2. Methods

### 2.1. Study Sample

**Figure 1:**
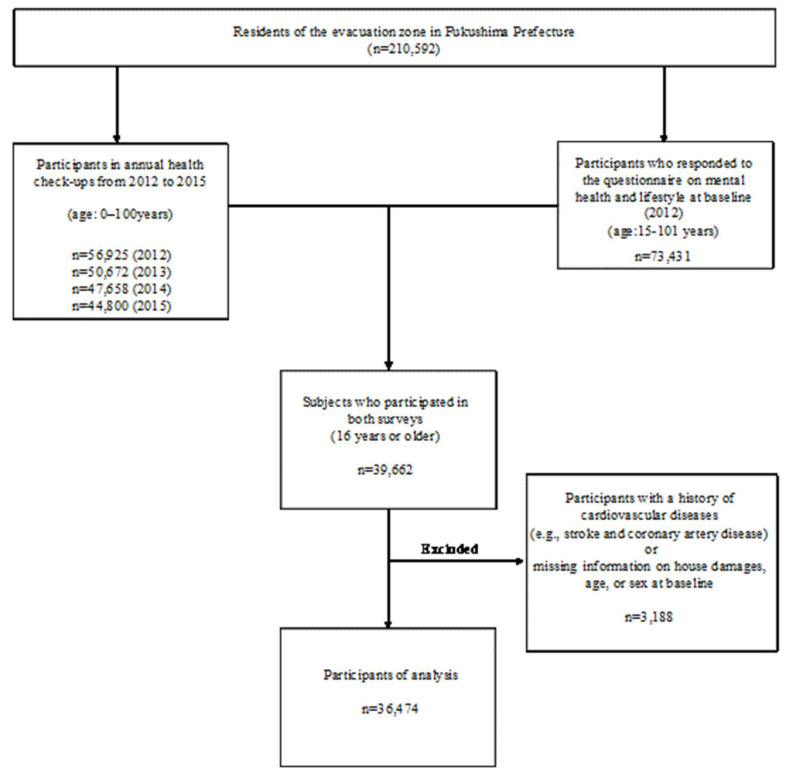
Flow chart of study participant selection.

Figure 1 shows a flowchart of study subject selection. The study participants were Japanese men and women living in communities near the Fukushima Daiichi Nuclear Power Plant in Fukushima Prefecture, including Tamura City (2010 Census population: 42,085), Minami-Soma City (71,661), Kawamata-machi (16,065), Hironomachi (5495), Naraha-machi (7927), Tomioka-machi (15,854), Kawauchi-mura (3074), Okuma-machi (11,553), Futaba-machi (7171), Namie-machi (21,551), Katsurao-mura (1582), and Iitate-mura (6584), with a total population of 210,592. Study participants were selected from those residents (total n = 210,592) who responded to the questionnaire on mental health and lifestyle conducted at the baseline in 2012 (n = 73,431; 34.9% response rate). Of these, only men and women (age: 16 years or older) who underwent comprehensive health checks in the Fukushima Health Management Survey from 2012 to 2015 were included (final n =39,662). The study protocol and baseline profiles are described in previous studies [17,18,19,20]. We excluded 3188 participants with a history of cardiovascular diseases (e.g., stroke and coronary artery disease) or missing information on house damages, age, or sex at baseline. As a result, 36,474 participants (14,941 men and 21,533 women) were eligible for the study from baseline, with at least one measurement of blood pressure levels between 2012 and 2015 following a clinical examination and no missing values for any covariates [21]. Follow-up examinations were conducted from 2012 to 2015 as part of the comprehensive health check in the Fukushima Health Management Survey. The questionnaire on mental health and lifestyle had been administered and the comprehensive health check had been conducted since January 2012. In this study, 2012 was defined as the baseline year, as it immediately followed the earthquake [18]. Furthermore, we separately analyzed participants with and without antihypertensive medication use between 2012 and 2015 using stratified analysis according to the baseline drinking status. Antihypertensive medication use was defined as “yes” in any annual survey between 2012 and 2015.

### 2.2. Questionnaire Survey on Living Environment Changes

In the questionnaire on mental health and lifestyle, we asked the participants “Where did you live after the earthquake?” to measure living environment changes. Respondents could choose one of six possible answers: “shelter”, “temporary housing”, “rented apartment”, “relative’s house”, “own house”, or “other”. In this study, people belonging to the categories of shelter, temporary housing, rented apartment, relative’s house, and other were together considered as “persons with living environment changes”. People belonging to the category of own house were regarded as “persons without living environment changes”; this category was used as a reference [22].

### 2.3. Blood Pressure Measurement

Blood pressure measurements in this study were carried out in accordance with the “Specific Health Checkup/Specific Health Guidance Guideline”, which is used in the specific health examinations conducted by the Ministry of Health, Labor and Welfare in Japan [23]. Specific health checkups are nationwide checkups for people aged 40 to 74 that focus on metabolic syndrome to prevent lifestyle-related diseases [23]. Details of the measurement method follow the guideline by the Japanese Society for Cardiovascular Disease Prevention [24]. Blood pressure levels were measured twice from the right arm of all participants after 1–2 min of rest in a seated position. We used averages of the first and second blood pressure measurements for each year from 2012 to 2015 for the analyses. Hypertension was defined as systolic blood pressure ≥ 140 mmHg, diastolic blood pressure ≥ 90 mmHg, or antihypertensive medication use. From baseline, 36,474 participants (14,941 men and 21,533 women) remained eligible for this study. The number of participants was 12,828 (35.2%) for all three subsequent annual follow-ups, 8088 (22.1%) for two follow-ups, 6518 (17.9%) for one follow-up, and 9040 (24.8%) for no follow-up. Therefore, 75.2% of the participants had two or more blood pressure measurements taken during the follow-up, and 97,652 blood pressure observations were used in the present analyses. 

### 2.4. Other Covariates

We calculated the body mass index (BMI) for all participants by dividing weight (kg) by height (m^2^). Socks and light clothing were included during height and weight measurement at baseline and follow-up. 

Each participant was interviewed to determine their usual weekly alcohol intake in “go” units, a traditional Japanese unit of volume equivalent to 23 g of ethanol. Information regarding smoking status and history; the present daily number of cigarettes smoked; and histories of hypertension, stroke, coronary heart disease, and antihypertensive medication use was also collected during the interview.

Low-density lipoprotein cholesterol (mg/dL), HDL cholesterol (mg/dL), triglycerides (mg/dL), blood glucose levels (mg/dL), and risk factors for cardiovascular diseases were measured at the annual health examination. Diabetes was defined as fasting glucose ≥ 7.0 mmol/L (126 mg/dL) or casual blood glucose ≥ 11.1 mmol (200 mg/dL) and/or on treatment for diabetes or HbA1c ≥ 6.5%. 

We used the Kessler 6 (K6) scale [25] for measuring nonspecific mental health distress in the participants. We asked participants if they had experienced any of the following six feelings during the past 30 days: “feeling so sad that nothing could cheer you up”, “feeling nervous”, “hopeless”, “restless or fidgety”, “feeling like everything was an effort”, and “feeling worthless” [26]. Each question was scored on a 5-point Likert-type scale from 0 to 4, with higher scores representing a worse mental health status (score range: 0–24). The Japanese version of the K6 scale has been professionally validated [27]. We defined nonspecific mental health distress as corresponding to a K6 score of ≥13 [26].

We used the question about sleep quality sourced from the Athens Insomnia Scale [28] for measuring participants’ sleep situation. We asked the question “Have you been satisfied with your sleep quality for the past month?” and offered the following response options: “all of the time”, “much of the time”, “a little”, or “almost never”. 

To assess whether participants performed regular physical exercises, we asked them “How often do you usually exercise?” and offered the following response options: “almost every day”, “2–4 days a week”, “1 day a week”, or “almost never”. This questionnaire was used in the specific health examinations conducted by the Ministry of Health, Labor and Welfare in Japan. Specific health checkups are nationwide checkups for people aged 40 to 74 that focus on metabolic syndrome to prevent lifestyle-related diseases [23]. Missing answers for variables were added into the models as dummy variables according to previous studies about linear mixed-effects models [29].

### 2.5. Ethical Approval

Informed consent from community representatives was obtained to conduct an epidemiological study based on the guidelines of the Council for International Organizations of Medical Science. This study was approved by the Ethics Committee of Fukushima Medical University (29064) and the Ethical Review Board of Juntendo University Faculty of Medicine (2017142). 

### 2.6. Statistical Analysis

*T*-tests and chi-square tests were used to compare sex-specific means and proportions of various lifestyle and psychological factors of baseline characteristics.

To examine the effect of living environment changes at baseline on changes in blood pressure over time, we analyzed data by using mixed-effects modeling, the widely accepted method for dealing with longitudinal data, which accounts for correlations among measurements for the same individual. In the present study, we used the following model (SAS MIXED procedure) [30]:

*Y_ij_* = (*β*_0_ + *b*_0i_) + *β_p_·*living environment changes *_i_* + *β_T_*·Time*_ij_* + *β_PT_* (living environment changes × time)*_ij_* + ε*_ij_*

where *Y_ij_* represents systolic and diastolic blood pressure levels for an individual *i* taken at time *j*, *β*_0_ is the overall intercept, *β_p_* is the effect of living environment changes considered as constant across time, *β_T_* represents the intercept and slope of the linear relationship between systolic and diastolic blood pressure levels and time at which the outcome was measured, and *β_PT_* is the effect of living environment changes on the slope describing the linear relationship between blood pressure levels and time (blood pressure changes per year). Model coefficients were estimated through maximum likelihood using the SAS MIXED procedure and specifying a compound symmetry structure for the covariance matrix [29,30,31,32].

Covariates included baseline age, delta age (difference in age between baseline and time at which the outcome was measured), smoking status (never, ex-smokers, or current smokers), drinking status (never, rarely, sometimes, or everyday), K6 score, subjective sleep, regular physical exercises, antihypertensive medication use from 2012 to 2015 (presence or absence of medication), and BMI (kg/m^2^). Covariate measurements were taken at baseline when living environment changes were assessed. When measurements were missing, we used the available measurements closest in time to when the living environment changes were assessed. To assess the potential modifying effects of baseline age and change in age on the relationship between changes in the living environment and blood pressure levels over time, we ran regression models that included cross-product terms for the interaction between age and change in age with living environment changes along with main effects. 

Furthermore, we separately analyzed participants with and without antihypertensive medication use between 2012 and 2015. Participants who had never used antihypertensive medication during the three years following baseline were included in the group without antihypertensive medication use. Those who used antihypertensive medication at baseline or during any follow-up surveys were included in the group with antihypertensive medication use. Because alcohol intake is a strong covariate for blood pressure levels [33], we conducted stratified analysis according to the baseline drinking status. 

We used SAS software version 9.4 (SAS Institute, Cary, NC, USA) for all analyses. A *p* value of < 0.05 (two-tailed) was considered statistically significant, except for *p* < 0.10 in the interaction analysis, according to previous studies [32,34].

## 3. Results

Table 1 shows sex-specific baseline characteristics according to living environment changes. Men and women experiencing these changes tended to be younger, and reported poor sleep quality and feelings of severe psychological distress. Men experiencing living environment changes were more likely to show increased mean BMI. Women experiencing living environment changes included a higher proportion of current smokers and drinkers.

Table 2 shows sex-specific changes in systolic and diastolic blood pressure levels over time according to living environment changes. In either sex at baseline, no significant difference in systolic blood pressure levels was observed after living environment changes. However, women who changed their living environment showed decreased systolic blood pressure levels (time-dependent difference [95% CI]: *β* = −0.20 mmHg [−0.37, −0.02]; *p* = 0.03), whereas men who changed their living environment showed no significant change in systolic blood pressure levels over time (time-dependent effect). 

Men with living environment changes had significantly higher diastolic blood pressure levels immediately after the earthquake than those without these changes (baseline effect [95% CI]: *β* = 0.63 mmHg [0.26, 1.00]; 77.3 mmHg vs. 77.8 mmHg; *p* < 0.001). No significant time-dependent effect was observed (time-dependent effect [95% CI]: *β* = 0.0001 mmHg [–0.15, 0.15]; *p* > 0.99). These results revealed that diastolic blood pressure levels, which increased after the earthquake, had not decreased over the three years in men who had living environment changes due to the earthquake. For women, diastolic blood pressure levels did not change at baseline or over the three years in any group.

Table 3 shows sex-specific changes in systolic and diastolic blood pressure levels over time according to living environment changes with and without antihypertensive medication use. Among men without antihypertensive medication use, those with living environment changes immediately after the earthquake had significantly higher systolic blood pressure (baseline effect [95% CI]: *β* = 1.11 mmHg [0.44, 1.79]; 125.1 mmHg vs. 126.3 mmHg; *p* = 0.001) and diastolic blood pressure levels (baseline effect [95% CI]: *β* = 0.87 [0.40, 1.35]; 76.2 mmHg vs. 76.9 mmHg; *p* < 0.001). Systolic blood pressure levels decreased after the three years (time-dependent effect [95% CI]: *β* = –0.30 mmHg; [–0.57, –0.03]; *p* = 0.03). However, no significant difference in diastolic blood pressure levels was observed during the follow-up period in men without antihypertensive medication use (time-dependent effect [95% CI]: *β* = –0.02 mmHg [–0.22, 0.18]; *p* = 0.84). 

Among men with antihypertensive medication use, those with living environment changes immediately after the earthquake had significantly lower systolic blood pressure levels (baseline effect [95% CI]: *β* = –0.97 mmHg [–1.75, –0.19]; 135.7 mmHg vs. 135.5 mmHg; *p* = 0.02) than men without living environment changes. However, no significant difference in diastolic blood pressure levels was observed at baseline and over the three years in men with antihypertensive medication use.

For women, systolic blood pressure levels decreased significantly over time, regardless of antihypertensive medication use (time-dependent effect [95% CI]: *β* = –0.30 mmHg; [–0.62, 0.01]; *p* = 0.06 for women with antihypertensive medication use; time-dependent effect [95% CI]: *β* = –0.27 mmHg; [–0.47, −0.06]; *p* = 0.01 for those without antihypertensive medication use). For women with antihypertensive medication use and living environment changes, diastolic blood pressure levels were significantly higher (baseline effect [95% CI]: *β* = 0.97 [0.47, 1.47] 76.8 mmHg vs. 78.0 mmHg; *p* < 0.001) immediately after the earthquake and decreased during the follow-up period (time-dependent effect [95% CI]: *β* = –0.24 mmHg [–0.46, –0.03]; *p* = 0.02). 

Sex-specific changes in systolic and diastolic blood pressure levels over time after living environment changes according to the baseline drinking status are shown in Supplemental Table S1. The associations between changes in the living environment and diastolic blood pressure levels immediately after the earthquake were found in men who were current drinkers, but not in women. No overtime effects of living environment changes on blood pressure levels were noted in both men and women.

Sex-specific baseline characteristics with only one baseline measurement according to living environment changes are shown in Supplemental Table S2. No significant difference was observed in baseline characteristics between the participants who had more than one blood pressure measurement (Table 1) and those with only one baseline measurement.

## 4. Discussion

In this study, men with changes in their living environment (i.e., shelter, temporary housing, rented apartment, or relative’s house) showed significantly higher diastolic blood pressure levels than those who continued to live in their own home at baseline. For men, the time-dependent effect of diastolic blood pressure levels associated with the living environment was not statistically significant during the study period, which indicated that the difference in diastolic blood pressure associated with living environment changes at baseline remained after three years. The effect of living environment changes on diastolic blood pressure increment was also evident in men without antihypertensive medication use and in those who were current drinkers at baseline.

Some possible mechanisms can explain the adverse effect of living environment changes due to the earthquake on blood pressure, for example, psychological distress and adverse lifestyle changes. A relationship between disasters and blood pressure levels has been documented in review articles based on the mechanisms and management of disaster hypertension [35]. Changes in the living environment following disasters cause psychological stress, including fear, anxiety, and depression. These factors also contribute to poor sleep quality and daytime physical inactivity [35]. Furthermore, a week or more after the disaster, high-salt foods, such as cup noodles, were delivered to residents in shelters, resulting in excessive salt consumption in daily eating habits [35]. Further, people on antihypertensive medications before the earthquake were forced to discontinue medications due to sudden changes in their living environment [35]. Particularly in Fukushima Prefecture, the effects of evacuation from the area around the Fukushima Daiichi Nuclear Power Plant have been prolonged. A study reported the increased prevalence of cardiovascular risk factors (i.e., hypertension, diabetes mellitus, and dyslipidemia) among community residents, and more so among evacuees from the evacuation zone of Fukushima Prefecture [15]. Because these disaster-specific factors and environments might induce disaster hypertension, psychological, social, and medical interventions are crucial for adequately controlling disaster hypertension and reducing the incidence of subsequent cardiovascular events in survivors [35]. 

The difference in time-dependent changes between systolic and diastolic blood pressure levels in this study may be due to the mechanisms and characteristics of increased blood pressure levels. An increase in systolic blood pressure levels is caused by age-related decreases in aortic compliance, while an isolated increase in diastolic blood pressure levels is caused by reduced peripheral vascular compliance. Isolated diastolic blood pressure increment is mostly noted in young and middle-aged people, male sex, those with poorer lifestyle practices (i.e., smoking, drinking, or physical inactivity), and those with hypercholesterolemia, hypertriglyceridemia, hyperglycemia, metabolic syndrome, and sleep apnea [36]. Isolated diastolic hypertension in younger generations is more likely to develop into systolic and diastolic hypertension or isolated systolic hypertension, thereby increasing cardiovascular mortality [37]. In this study, men with living environment changes were relatively younger (58.2 years for persons with living environment changes vs. 63.4 years for those without living environment changes; *p* < 0.001). As we found in the present study, observing and treating diastolic blood pressure in the younger generation of people who have recently experienced sudden living environment changes due to disasters is vital for reducing the risk of cardiovascular events. 

Studies have found various impacts of the Great East Japan Earthquake on residents and evacuees who have changed their housing type, such as shelters and temporary housing. These studies have reported significant differences in motor function [38], physical activity [39], body weight [40], and metabolic syndrome before and after the earthquake [41]. Furthermore, our previous study reported an increase in mean blood pressure levels and hypertension prevalence immediately after the earthquake, especially among male evacuees, due to the nuclear accident [15]. However, our current longitudinal study revealed that diastolic blood pressure, which increased after the earthquake, showed no decrease over the three years in the younger men. Our study is the first to reveal a longitudinal association between changes in the living environment and blood pressure increase. 

We found no significant association between living environment changes and blood pressure changes in women. Women with living environment changes were relatively younger than men (55.0 years vs. 58.2 years). Women had lower mean systolic (123.5 mmHg vs. 128.8 mmHg) and diastolic blood pressure levels (73.4 mmHg vs. 77.4 mmHg) at baseline than men, which made detecting any adverse effect of living environment changes on blood pressure difficult. Moreover, the proportion of respondents with problem drinking, calculated using the CAGE questionnaire [42], was 20.5% for men and 10.5% for women [20]. This figure was particularly high for men immediately after the earthquake, as found in our previous study [20]. A study conducted in the second year after the disaster has suggested that the risk factors of postdisaster problem drinking consisted of being male, less than 65 years old, experiencing sleep insufficiency or psychological distress, and drinking heavily [43]. Changes in lifestyle and mental distress associated with living environment were more frequent in men than women, which may have led to the effect of living environment changes on blood pressures being more evident for men than women. 

We considered that mental stress due to the earthquake and aging may also increase systolic blood pressure over time. However, both men and women who had changed their living environments and were not using antihypertensive medications tended to exhibit decreased systolic blood pressure over time. One explanation is that the mean age of persons (both men and women) with living environment changes was significantly lower than that of persons without living environment changes. Furthermore, we did not consider further changes in the living environment, negative emotional status, and lifestyles from 2012 to 2015 in our analyses. Housing prospects for persons in temporary housing were associated with psychological distress one year after the earthquake [44]. These may have affected changes in systolic blood pressure levels over time following the earthquake, which could have made detecting any effect of living environment changes on blood pressure changes difficult. Moreover, information regarding blood pressure levels was only available for the three years following the earthquake. We could not rule out that participants with living environment changes may have had high systolic blood pressure levels before the earthquake, which may have affected our results.

A strength of the present study is a prospective design with a large sample size to detect the association between changes in the living environment and blood pressure levels over the three years following the Great East Japan Earthquake. Most participants (75.2%) had more than one blood pressure measurement. Moreover, no significant difference was observed in baseline characteristics between the participants who had more than one blood pressure measurement and those with only one baseline measurement (Supplemental Table S2). Therefore, withdrawal from the follow-up was unlikely to affect the results.

The present study has several limitations. First, the proportion of participants who responded to the questionnaire on mental health and lifestyle conducted at the baseline in 2012 was 34.9%, and the remaining 65.1% of residents who did not respond could not be considered. Second, this study did not evaluate socioeconomic factors other than living environment changes as potential confounders. We did not collect information about other environmental or socioeconomic factors, such as education status, job status, and marital status, so we could not rule out the possibility of residual confounding factors. Third, we had no information on the salt intake of participants. After the disaster, foods containing salt, such as cup noodles, were delivered to residents in shelters, which resulted in excessive salt intake among daily eating habits [35]. However, we could not assess the relationship between salt intake and blood pressure due to changes in dietary content after the earthquake. Finally, the comprehensive health check-related covariates were included during the follow-up, but we only had information about living environment changes, the K6 score, regular physical exercise, and subjective sufficient sleep at baseline. Therefore, we could not consider further incorporation of all these changes as time-varying covariates from 2012 to 2015. 

## 5. Conclusions

In conclusion, the present longitudinal observational study showed that sudden living environment changes caused by the earthquake led to increased diastolic blood pressure levels immediately after the earthquake among middle-aged men. The increased diastolic blood pressure did not subsequently decrease in men during the three years following the Great East Japan Earthquake. The effect of living environment changes on diastolic blood pressure increase was also evident in men without antihypertensive medication use during the study period and in men with a current drinker status at baseline. Thus, the sudden change in the living environment after the disaster incurred long-term effects on increased diastolic blood pressure. By 2021, 10 years had passed since the Great East Japan Earthquake, and while reconstruction is in progress, many Fukushima residents (almost 40,000) are still living in changed environments due to the nuclear power plant accident. Continuous antihypertensive treatment, active blood pressure monitoring after the disaster, and interventions consisting of antihypertensive medications are pivotal for adequately controlling blood pressure among residents. Along with hypertension management, psychological interventions and early housing prospects are considered crucial and useful for preventing high systolic and diastolic blood pressure levels, as well as for controlling mental stress, especially in the younger generation. 

## Figures and Tables

**Table 1:** Sex-specific baseline characteristics by changes in living environment (n = 36,474).

	Men			Women		
	Without Living Environment Changes	With Living Environment Changes	*p* for Difference	Without Living Environment Changes	With Living Environment Changes	*p* for Difference
Number	4518	10,423		5872	15,661	
Age, mean (SD)	63.4 (14.1)	58.2 (16.4)	<0.001	60.9 (15.0)	55.0 (16.9)	<0.001
BMI (kg/m^2^), mean (SD)	23.9 (3.3)	24.3 (3.4)	<0.001	23.3 (3.7)	23.2 (3.9)	0.27
Systolic blood pressure, mean (SD)	130.0 (16.2)	128.8 (15.5)	<0.001	126.7 (17.0)	123.5 (17.1)	<0.001
Diastolic blood pressure, mean (SD)	76.8 (10.6)	77.4 (10.5)	0.002	74.0 (10.5)	73.4 (10.7)	<0.001
Antihypertensive medication use, %	26.9	24.0	<0.001	26.1	19.4	<0.001
Hypertension, %	45.5	41.5	<0.001	40.1	31.3	<0.001
High triglycerides, %	21.6	25.8	<0.001	14.5	14.3	0.69
High LDL cholesterol, %	35.0	38.1	<0.001	47.8	44.1	<0.001
Low HDL cholesterol, %	24.9	24.9	0.95	27.2	22.5	<0.001
Diabetes, %	16.8	15.8	0.14	9.3	7.8	<0.001
Smoking Status:						
never, %	30.9	28.5	0.003	86.7	79.2	<0.001
ex-smokers, %	42.5	39.2	<0.001	4.6	7.4	<0.001
current smokers, %	23.2	27.2	<0.001	4.0	8.4	<0.001
Drinking Status:						
never, %	33.1	34.5	0.09	76.7	72.5	<0.001
Occasional drinkers, %	23.6	22.5	0.15	17.3	18.4	0.07
current drinkers, %	42.6	41.4	0.19	4.8	7.7	<0.001
K6 ≥ 13, %	6.2	12.8	<0.001	10.2	17.5	<0.001
Regular physical exercise (yes), %	60.0	55.3	<0.001	54.4	47.7	<0.001
Subjective sufficient sleep (yes), %	93.1	84.0	<0.001	89.9	78.9	<0.001
Hypertension: systolic pressure ≥ 140 mmHg or diastolic pressure ≥ 90 mmHg, and/or antihypertensive medication use						
High triglycerides: triglycerides ≥ 150 mg/dL (1.69 mmol/L)						
High LDL cholesterol: LDL cholesterol ≥ 140 mg/dL (3.62 mmol/L) or medication use						
Low HDL cholesterol: HDL cholesterol < 40 mg/dL (1.03 mmol/L) or medication use						
Diabetes: fasting glucose ≥ 126 mg/dL (7.0 mmol/L), or casual blood glucose ≥ 200 mg/dL (11.1 mmol) and/or on treatment or HbA1c ≥ 6.5% Drinking Status: never drinkers: never or rarely, occasional drinkers: sometimes, current drinkers: everyday						

**Table 2:** Sex-specific changes in systolic and diastolic blood pressures with time by changes in living environment.

	Men			Women		
	Without Living Environment Changes	With Living Environment Changes		Without Living Environment Changes	With Living Environment Changes	
Number	4518	10,423		5872	15,661	
	Reference	*β* (95% CI)	*p* value	Reference	*β* (95% CI)	*p* value
Systolic blood pressure						
Effect at baseline	0	0.09 (−0.43, 0.61)	0.73	0	−0.32 (−0.76, 0.12)	0.15
Time-dependent effect **	0	−0.07 (−0.28, 0.15)	0.55	0	−0.20 (−0.37, −0.02)	0.03 *
Mean value for 2012 (SD)	128.7 (0.3)	129.3 (0.2)		124.3 (0.2)	124.4 (0.1)	
Mean value for 2015 (SD)	128.9 (0.3)	129.1 (0.2)		125.3 (0.2)	124.6 (0.1)	
Diastolic blood pressure						
Effect at baseline	0	0.63 (0.26, 1.00)	<0.001 *	0	0.22 (−0.09, 0.53)	0.16
Time-dependent effect **	0	0.0001 (−0.15, 0.15)	>0.99	0	0.02 (−0.11, 0.14)	0.79
Mean value for 2012 (SD)	77.3 (0.2)	77.8 (0.1)		73.7 (0.1)	74.0 (0.1)	
Mean value for 2015 (SD)	75.7 (0.2)	76.4 (0.1)		72.7 (0.2)	73.3 (0.1)	
* *p* value of interaction with time						
** Time × changes in living environment						
Adjusted for age, antihypertensive medication use from 2012 to 2015, current smoking, drinking status, regular physical exercise, subjective sufficient sleep, K6 score, and BMI from 2012 to 2015. The *p*-value of interaction with time is based on the assessment comparing between reference category (“without living-environment changes”) and other categories by using the linear mixed-effect models.						

**Table 3:** Sex-specific changes in systolic and diastolic blood pressures with time by changes in living environment with and without antihypertensive medication use.

	Men			Women		
	Without Living Environment Changes (Reference)	With Living Environment Changes		Without Living Environment Changes (Reference)	With Living Environment Changes	
	No antihypertensive medication users					
Number	1798	4508		2730	7995	
		*β* (95% CI)	*p* value		*β* (95% CI)	*p* value
Systolic blood pressure						
Baseline difference	0	1.11 (0.44, 1.79)	0.001 *	0	−0.03 (−0.56, 0.51)	0.92
Time-dependent difference **	0	−0.30 (−0.57, −0.03)	0.03 *	0	−0.27 (−0.47, −0.06)	0.01 *
Mean value for 2012 (SD)	125.1 (0.3)	126.3 (0.2)		120.3 (0.3)	120.5 (0.1)	
Mean value for 2015 (SD)	126.0 (0.3)	126.2 (0.2)		121.1 (0.3)	120.6 (0.2)	
Diastolic blood pressure						
Baseline difference	0	0.87 (0.40, 1.35)	<0.001 *	0	0.10 (−0.27, 0.48)	0.58
Time-dependent difference **	0	−0.02 (−0.22, 0.18)	0.84	0	0.08 (−0.07, 0.23)	0.30
Mean value for 2012 (SD)	76.2 (0.2)	76.9 (0.1)		72.2 (0.2)	72.4 (0.1)	
Mean value for 2015 (SD)	75.7 (0.2)	76.5 (0.2)		71.6 (0.2)	72.3 (0.1)	
	Antihypertensive medication users					
Number	1216	2504		1535	3042	
		*β* (95% CI)	*p* value		*β* (95% CI)	*p* value
Systolic blood pressure						
Baseline difference	0	−0.97 (−1.75, −0.19)	0.02 *	0	−0.29 (−1.02, 0.45)	0.45
Time-dependent difference **	0	0.18 (−0.16, 0.52)	0.30	0	−0.30 (−0.62, 0.01)	0.06 *
Mean value for 2012 (SD)	135.7 (0.4)	135.5 (0.3)		134.5 (0.3)	134.8 (0.2)	
Mean value for 2015 (SD)	132.6 (0.4)	132.7 (0.2)		133.1 (0.3)	132.2 (0.2)	
Diastolic blood pressure						
Baseline difference	0	0.46 (−0.08, 1.00)	0.10	0	0.97 (0.47, 1.47)	<0.001 *
Time-dependent difference **	0	−0.01 (−0.24, 0.22)	0.92	0	−0.24 (−0.46, −0.03)	0.02 *
Mean value for 2012 (SD)	79.0 (0.3)	79.4 (0.2)		76.8 (0.2)	78.0 (0.2)	
Mean value for 2015 (SD)	76.0 (0.2)	76.6 (0.2)		75.0 (0.2)	75.4 (0.2)	
* *p* value of interaction with time						
** Time × changes in living environment						
Adjusted for age, antihypertensive medication use from 2012 to 2015, current smoking, drinking status, regular physical exercise, subjective sufficient sleep, K6 score, and BMI from 2012 to 2015. The p-value of interaction with time is based on the assessment comparing between reference category (“without living environment changes”) and other categories by using the linear mixed-effect models.						

## Data Availability

The datasets analyzed during the present study are not publicly available because the data from the Fukushima Health Management Survey belongs to the government of Fukushima Prefecture and can only be used within the organization.

## Supplementary Materials

The following supporting information can be downloaded at: https://www.mdpi.com/article/10.3390/ijerph20010857/s1, Table S1: Sex-specific changes in systolic and diastolic blood pressures with time by changes in living environment in everyday life according to drinking status; Table S2: Sex-specific baseline characteristics of participants with only one baseline measurement by changes in living environment (n = 9040).
