# Supplementary material for: Longitudinal Trends in Blood Pressure Associated with The Changes in Living Environment Caused by the Great East Japan Earthquake: The Fukushima Health Management Survey
Source: Int J Environ Res Public Health. 2023 Jan 3;20(1):857. doi: 10.3390/ijerph20010857 (PMC9819706; doi:10.3390/ijerph20010857)
Supplement: Supplementary file 1 [file ijerph-20-00857-s001.zip › ijerph-2071075-supplementary.pdf]

International Journal of Environmental Research and Public Health

MDPI

Supplementary Table S1. Sex-specific changes in systolic and diastolic blood pressures with time by changes in living environment in everyday life according to drinking status.

	Men			Women		
	**Without living environment changes (reference)**	**With living environment changes**		**Without living environment changes (reference)**	**With living environment changes**	
	Non-current drinkers					
**Number**	1,711	3,925		4,058	10,072	
		β (95% CI)	*p* value		*β* (95% CI)	*p* value
**Systolic blood pressure**						
Baseline difference	0	0.14 (-0.54, 0.81)	0.69	0	-0.29 (-0.74, 0.16)	0.20
Time-dependent difference**	0	-0.19 (-0.47, 0.10)	0.20	0	-0.22 (-0.40, -0.04)	0.02*
**Diastolic blood pressure**						
Baseline difference	0	0.49 (0.01, 0.97)	0.045	0	0.23 (-0.08, 0.54)	0.15
Time-dependent difference**	0	0.04 (-0.16, 0.24)	0.70	0	0.02 (-0.11, 0.14)	0.78
	Current drinkers of ethanol at 1–22 g/day or ≥23 g/day					
**Number**	1,303	3,087		207	965	
		β (95% CI)	*p* value		*β* (95% CI)	*p* value
**Systolic blood pressure**						
Baseline difference	0	-0.03 (-0.80, 0.75)	0.95	0	0.43 (-1.30, 2.15)	0.63
Time-dependent difference**	0	0.13 (-0.20, 0.47)	0.43	0	-0.66 (-1.46, 0.14)	0.10
**Diastolic blood pressure**						
Baseline difference	0	0.72 (0.17, 1.27)	0.01*	0	0.74 (-0.53, 2.01)	0.25
Time-dependent difference**	0	0.005 (-0.23, 0.24)	0.97	0	-0.38 (-0.96, 0.21)	0.21

* *p* value of interaction with time

** Time×changes in living environment

Adjusted for age, antihypertensive medication use from 2012 to 2015, current smoking, drinking status, regular physical exercise, subjective sufficient sleep, K6 score, and BMI from 2012 to 2015.

The p-value of interaction with time is based on the assessment comparing between reference category ("without living-environment changes") and other categories by using the linear mixed-effect models.

Supplemental Table S2. Sex-specific baseline characteristics of participants with only one baseline measurement by changes in living environment (n=9,040).

	Men			Women		
	**Without living environment changes**	**With living environment changes**	***p* for difference**	**Without living environment changes**	With living environment changes	*p* for difference
**Number**	1,157	2,741		1,384	3,758	
**Age, mean (SD)**	59.5 (17.4)	53.6 (18.5)	<0.001	58.1 (18.4)	50.9 (19.4)	<0.001
**BMI (kg/m^2), mean (SD)**	24.1 (3.7)	24.3 (3.8)	0.08	23.3 (3.8)	23.2 (4.2)	0.39
**Systolic blood pressure, mean (SD)**	129.8 (16.7)	127.4 (15.7)	<0.001	126.4 (17.8)	121.7 (17.8)	<0.001
**Diastolic blood pressure,mean (SD)**	76.5 (10.9)	76.6 (10.9)	0.67	73.4 (11.1)	72.3 (11.3)	0.01
**Antihypertensive medication use, %**	15.9	13.3	0.031	15.6	10.4	<0.001
**Hypertension, %**	38.1	33.2	0.003	33.2	24.3	<0.001
**High triglycerides, %**	24.0	26.7	0.09	15.2	14.8	0.75
**High LDL cholesterol, %**	33.8	35.2	0.41	42.4	38.9	0.02
**Low HDL cholesterol, %**	23.0	22.2	0.60	22.9	18.5	<0.001
**Diabetes, %**	17.9	15.8	0.10	11.5	8.3	<0.001
**Smoking Status:**						
**never, %**	28.1	25.5	0.09	81.1	72.9	<0.001
**ex-smokers, %**	39.1	34.5	0.006	6.4	8.7	0.008
**current smokers, %**	28.3	34.1	<0.001	5.3	11.5	<0.001
**Drinking Status:**						
**never, %**	36.4	36.7	0.85	76.0	70.9	<0.001
**Occasional drinkers, %**	22.9	22.8	0.94	16.7	18.3	0.18
**current drinkers, %**	38.6	37.8	0.64	5.3	8.2	<0.001
**K6≥13, %**	6.7	13.8	<0.001	10.7	16.9	<0.001
**Regular physical exercise (yes), %**	56.4	48.6	<0.001	47.1	42.0	0.001
**Subjective sufficient sleep (yes), %**	91.2	83.6	<0.001	89.9	79.2	<0.001

Hypertension: systolic pressure ≥ 140mmHg or diastolic pressure ≥ 90mmHg, and/or antihypertensive medication use

High triglycerides: triglycerides ≥ 150 mg/dl (1.69mmol/l)

High LDL cholesterol: LDL cholesterol ≥ 140mg/dl (3.62mmol/l) or medication use

Low HDL cholesterol:HDL cholesterol < 40mg/dl (1.03mmol/l) or medication use

Diabetes: fasting glucose ≥ 126mg/dl (7.0mmol/l), or casual blood glucose ≥ 200mg/dl (11.1mmol) and/or on treatment or HbA1c ≥ 6.5%

Drinking Status: never drinkers: never or rarely, occasional drinkers: sometimes, current drinkers ：everyday
